# Editorial: Bone metastases and secondary osteoporosis

**DOI:** 10.3389/fendo.2024.1429246

**Published:** 2024-05-16

**Authors:** Qiang Huo, Shan Wang, Lihong Guo, Yining Shen, Reginald M. Gorczynski, Qiaoli Zhai, Liang Li, Lei Zhang, Tao Li

**Affiliations:** ^1^Clinical Research Center, Nanjing Jiangbei Hospital, Nanjing, China; ^2^Center for Translational Medicine, Zibo Central Hospital, Zibo, China; ^3^Department of Rehabilitation Medicine, Air Force Medical Center, Air Force Medical University, PLA, Beijing, China; ^4^Department of Otolaryngology, Zibo Central Hospital, Zibo, China; ^5^College of Integrated Traditional Chinese and Western Medicine, Jining Medical University, Jining, China; ^6^Department of Surgery & Immunology, University of Toronto, Toronto, ON, Canada; ^7^Department of Spine Surgery, Zibo Central Hospital, Zibo, China; ^8^Department of Stomatology, Zibo Central Hospital, Zibo, China; ^9^Orthopedics Center, Nanjing Jiangbei Hospital, Nanjing, China

**Keywords:** bone metastases, osteoporosis, periodontitis, breast cancer, survival analysis

Bone metastasis (BM) is one of the most common complications of malignancies, and axial bone is reported as the most frequent location of metastasis (1). Bone is a dynamic tissue, and bone remodeling is crucial not only to replace primary with secondary bones, but also to repair skeleton microdamage and ensure normal calcium homeostasis (2). Osteoporosis is characterized by deterioration of bone density and architecture, along with increased fracture risk, which is correlated with the imbalanced activation of bone formation and bone resorption that closely related to the biological activities of osteoblasts and osteoclasts (3). On the other hand, both osteoporosis and periodontitis, a periodontal disease that represents a chronic inflammation of the periodontal tissue and alveolar bone, are inflammation-driven, age-related bone disorders, and recent studies have provided compelling evidence to support the correlation between systemic and alveolar bone loss. The mechanistic links, shared risk factors, and therapeutic implications have been investigated in both diseases, and age-related oxidative stress and senescence are considered underlying mechanisms that cause the uncoupling of bone remodeling process (4).

Skeletal-related events (SREs) usually refer to major complications of tumor bone disease such as pathological fracture, spinal cord compression, hypercalcemia, the need for radiotherapy and/or surgery, and the like (5). Since osteoblasts and osteoclasts play important roles in bone remodeling, which is highly similar to the inflammatory process, the interactions between osteoblasts, osteoclasts, malignant cells and immune cells are considered crucial for bone metastasis (6). Secondary osteoporosis refers to osteoporosis caused by diseases, drug treatment, or other reasons. The main clinical symptoms include osteopenia, bone microstructure changes, increased bone fragility and easy fracture (7). Bone mineral density (BMD) has been recognized as the gold standard for diagnosis of osteoporosis. In addition, trabecular bone score (TBS), or TBS in combination with BMD, have also been shown clinically effective for osteoporosis and fracture management (8). Previous studies have suggested that bone metastasis is an important factor causing secondary osteoporosis, which is associated with decreased quality of life and increased medical costs, adding great medical burdens to both patients and our society. The treatment strategies of secondary osteoporosis caused by various malignancies are dissimilar, but most of them involve the application of intravenous or oral bisphosphonates, and denosumab (9, 10).

Breast cancer (BC) is one of the most diagnosed female cancers, and bone metastases usually occurred in breast cancer patients. Whether palliative radiotherapy could improve the prognosis of metastatic patients remains unclear. Li et al. collected 488 metastatic breast cancer patients with human epidermal growth factor receptor 2 (HER2) positive diagnosis and anti-HER2 targeted therapy, to explore the clinical efficacy of palliative local radiotherapy in these patients. This observational study demonstrated that patients in the radiotherapy group got better overall survival (OS) than those in the non-radiotherapy group, and propensity score matching (PSM) analysis also confirmed the prognostic superiority in 135 pairs of baseline-matched cases. On the other hand, the authors also identified another two predictive factors, brain and liver metastasis, associated with OS in HER2 positive breast cancer patients using multivariate Cox regression models. Their research indicated that local radiotherapy was associated with longer survival time in patients who were diagnosed with HER2+ metastatic breast cancer and treated with anti-HER2 therapy.

Despite being the mainstay of hormonal therapy (HT) in post-menopausal breast cancers, aromatase inhibitors (AIs) which block the hormone-mediated growth signals are considered detrimental to bone health, inducing cancer treatment-induced bone loss (CTIBL) and leading to secondary osteoporosis. Another observational clinical study conducted by Galvano et al. retrospectively identified fifty early-stage BC patients with osteopenia and estrogen and/or progesterone receptor-positive (ER+ and/or PR+) diagnoses. Those cases were treated with AIs to prevent progression and metastasis, and bone modifying agents (BMAs) including denosumab or alendronate to prevent secondary osteoporosis. The authors demonstrated that the application of BMAs such as alendronate could significantly improve the BMD then preventing secondary osteoporosis at the lumbar spine levels, and impact pain control, recommending alendronate as an option in these frail BC settings.

Innovative tools were also included in our Research Topic. The necessity of post-mastectomy radiotherapy (PMRT) for patients with T_1-2_N_1_M_0_ breast cancer remains controversial. Jin et al. trained and developed two machine learning survival models using a training dataset including 35,347 patients with HR+/HER2- early-stage breast cancer who had received mastectomy. Based on their deep learning neural network model, the authors identified key factors relating to patients’ overall survival, including age, tumor size, positive regional nodes, and so forth. Meanwhile, the results also suggested that patients with tumor size over 14 mm and age older than 54 and the cohort with tumor size over 14 mm and grade worse than well-differentiated could benefit from the PMRT. The authors also developed a Cloud-based recommender system for PMRT and deployed it on the Internet (https://github.com/snowflake-Zhao/BRCA-I-PMRT), serving as a valuable tool when finding the most suitable adjuvant therapeutic strategy for BC patients.

Whether primary tumor resection (PTR) should be recommended for BC patients with synchronous BM remains unclear. In Tong’s study, 5,625 BC patients with BM were identified using the Surveillance, Epidemiology, and End Results (SEER) database, and were used to establish a visualized model for identifying optimal candidates for aggressive locoregional surgical treatment. Kaplan-Meier (K-M) survival and Cox regression analyses revealed that local surgery provided better survival in most subgroups and metastatic patterns. Surgery-benefit-related factors, including T stage, radiotherapy, race, liver metastasis, brain metastasis, and breast subtype, were identified by Logistic regression models and combined to establish the nomogram and an online tool for identifying optimal surgical candidates (probability calculator, https://sunshine1.shinyapps.io/DynNomapp/), confirming the prognostic significance of aggressive locoregional surgery in BC patients with BM.

In this Research Topic, we provided a forum for discussion on the relationship between bone metastasis and secondary osteoporosis. While collecting, we emphasized more on original research, and reviewed and collected excellent original works, which have advanced current knowledge both clinically and laboratorially. Understanding the association between these diseases and their interplay calls for more well-controlled studies to explore their mechanistic links, interdisciplinary management, and potential therapeutics.

## Author contributions

QH: Data curation, Formal analysis, Funding acquisition, Investigation, Supervision, Writing – original draft. SW: Data curation, Formal analysis, Investigation, Supervision, Writing – original draft. LG: Data curation, Formal analysis, Methodology, Writing – review & editing. YS: Data curation, Formal analysis, Methodology, Writing – review & editing. RG: Conceptualization, Methodology, Supervision, Writing – review & editing. QZ: Funding acquisition, Methodology, Supervision, Writing – review & editing. LL: Project administration, Resources, Supervision, Writing – review & editing. LZ: Project administration, Resources, Supervision, Writing – review & editing. TL: Methodology, Project administration, Resources, Supervision, Writing – review & editing.
